# DCTPP1 prevents a mutator phenotype through the modulation of dCTP, dTTP and dUTP pools

**DOI:** 10.1007/s00018-019-03250-x

**Published:** 2019-08-03

**Authors:** Blanca Martínez-Arribas, Cristina E. Requena, Guiomar Pérez-Moreno, Luis M. Ruíz-Pérez, Antonio E. Vidal, Dolores González-Pacanowska

**Affiliations:** 1 Instituto de Parasitología y Biomedicina “López-Neyra”, Consejo Superior de Investigaciones Científicas (CSIC), Parque Tecnológico de Ciencias de la Salud, Avenida del Conocimiento, 17, 18016 Armilla, Granada Spain; 2 Present Address: MRC London Institute of Medical Sciences, Du Cane Road, London, W12 0NN UK; 3 Present Address: Institute of Clinical Sciences, Imperial College London, Du Cane Road, London, W12 0NN UK

**Keywords:** DCTPP1, DNA damage, Pyrimidine salvage, Pyrimidine homeostasis, dNTP pool

## Abstract

To maintain dNTP pool homeostasis and preserve genetic integrity of nuclear and mitochondrial genomes, the synthesis and degradation of DNA precursors must be precisely regulated. Human all-alpha dCTP pyrophosphatase 1 (DCTPP1) is a dNTP pyrophosphatase with high affinity for dCTP and 5′-modified dCTP derivatives, but its contribution to overall nucleotide metabolism is controversial. Here, we identify a central role for DCTPP1 in the homeostasis of dCTP, dTTP and dUTP. Nucleotide pools and the dUTP/dTTP ratio are severely altered in DCTPP1-deficient cells, which exhibit an accumulation of uracil in genomic DNA, the activation of the DNA damage response and both a mitochondrial and nuclear hypermutator phenotype. Notably, DNA damage can be reverted by incubation with thymidine, dUTPase overexpression or uracil-DNA glycosylase suppression. Moreover, DCTPP1-deficient cells are highly sensitive to down-regulation of nucleoside salvage. Our data indicate that DCTPP1 is crucially involved in the provision of dCMP for thymidylate biosynthesis, introducing a new player in the regulation of pyrimidine dNTP levels and the maintenance of genomic integrity.

**Electronic supplementary material:**

The online version of this article (10.1007/s00018-019-03250-x) contains supplementary material, which is available to authorized users.

## Introduction

The accuracy of DNA replication is essential for the maintenance of genome integrity. The absolute and relative concentrations of the four deoxyribonucleoside triphosphates (dNTPs) are key determinants of the fidelity of DNA replication [1, 2]. As a result, an imbalanced dNTP pool can strongly promote replication errors [3, 4]. On the other hand, low dNTP availability causes mitochondrial and chromosomal instability in eukaryotic cells, especially after DNA damage is inflicted, since repair mechanisms require DNA synthesis [5]. Therefore, a tight control of the biosynthetic and catabolic pathways that regulate dNTPs levels is critical, since defects in their regulation originates replication stress and compromises genome stability [6–8].

Among other activities, the metabolic breakdown of nucleotide or nucleosides may involve phosphorylases, nucleotidases, NTP pyrophosphatases and triphosphohydrolases. In addition, enzymatic hydrolysis of damaged nucleotides cleanses the DNA precursor pool by eliminating potentially mutagenic modified nucleotides. Such is the case of the human enzyme MTH1 responsible for the elimination of 8-oxo-dGTP [9]. In this respect, the recently characterized human all-α NTP pyrophosphatase DCTPP1 (dCTP pyrophosphatase 1) catalyzes the hydrolysis of dCTP into dCMP and pyrophosphate, a conversion which has been proposed to keep the cellular dCTP pool balanced [10]. DCTPP1 can also hydrolyze in vitro C5-modified dNTPs such as 5-halogenated, 5-methyl and 5-formyl deoxycytidine, and therefore may have an additional ‘house-cleaning’ function. We have previously reported that DCTPP1-deficient cells accumulate high levels of dCTP and are hypersensitive to exposure to the nucleoside analogs 5-iodo-2′-deoxycytidine and 5-methyl-2′-deoxycytidine [10]. In addition, DCTPP1 counteracts the cytotoxic effect of the antitumoral demethylating agent decitabine (5-aza-deoxycytidine) by removing 5-aza-dCTP from the nucleotide pool and is being studied as a potential drug target to improve decitabine-based chemotherapy [11, 12]. In contrast with the role initially proposed for DCTPP1, a study performed by Song et al. supports the notion that the role of the enzyme is to regulate intracellular levels of methyl-dCTP, a function that directly impacts on the degree of DNA methylation [13]. This surprising observation is in conflict with a study performed by Zauri et al. [14], showing that due to strict substrate specificity of nucleotide salvage enzymes, newly synthesized DNA is protected against the incorporation of epigenetically modified forms of cytosine that are not present in the dNTP pool.

DCTPP1, which localizes to the nucleus, cytosol and mitochondria in human cells [10], is highly expressed in embryonic and proliferative tissues with an expanded nucleotide pool (Human Protein Atlas, www.proteinatlas.org). The enzyme is also up-regulated in multiple human carcinomas and cancer stem cells [13, 15, 16]. Together, these observations strongly point toward an important role of DCTPP1 in the homeostasis of the dNTP pool in highly proliferative cells with a great demand for DNA precursors.

In the present study, we examine the role of DCTPP1 in controlling pyrimidine nucleotide homeostasis in different cell types. We show that strong down-regulation of DCTPP1 results in an increased dCTP pool, reduced levels of dTTP and the appearance of a potentially genotoxic pool of dUTP. As a result, DCTPP1-deficient cells are more prone to uracil misincorporation and exhibit an activated DNA damage response (DDR), altered cell cycle progression and a mutator phenotype that affects both chromosomal and mitochondrial DNA (mtDNA). Furthermore we demonstrate that in the absence of DCTPP1, cells are highly dependent on nucleoside salvage for provision of dTTP. Taken together, our findings support a prominent role for DCTPP1 in dTTP de novo synthesis and in the balance of canonical pyrimidine nucleotide pools, a metabolic function that is intrinsically linked to the preservation of genome integrity. We propose that the function of this nucleotidohydrolase should be taken into account when studying the underlying causes of diseases involving an imbalance of the dNTP pools and the mode of action of anticancer analogs.

## Results

### Down-regulation of DCTPP1 expression impairs proliferation and perturbs the dNTP pool of MCF-7 cells

**Fig. 1:**
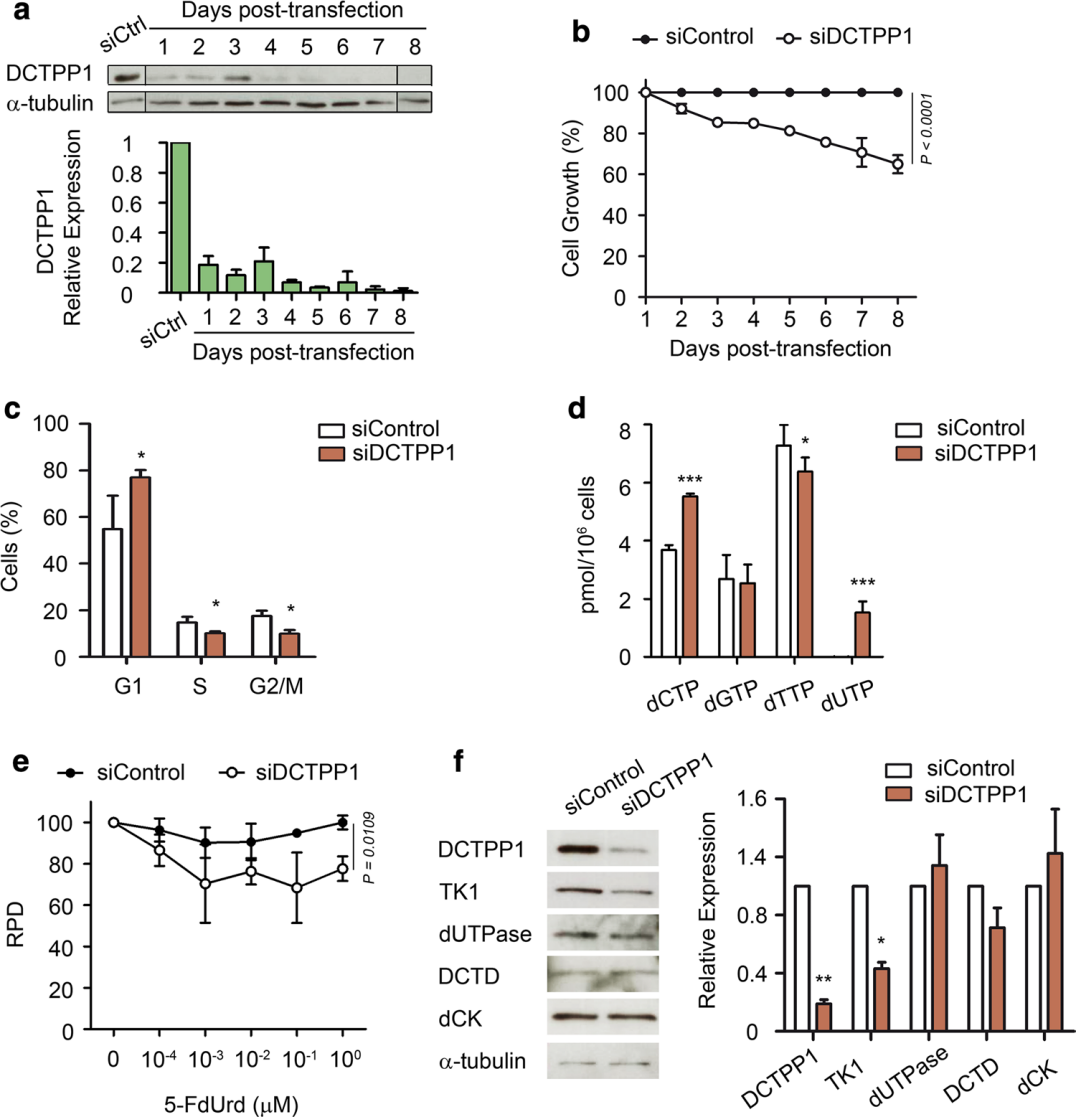
DCTPP1 depletion impairs proliferation and alters the composition of the dNTP pool of MCF-7 cells. **a** Western blot analysis of MCF-7 cells transfected with DCTPP1 and control (siCtrl) siRNAs. Anti-α-tubulin was used to normalize protein levels. The image was cropped and edited for easier comparison. Values are mean ± SD (*n *≥ 3). **b** Proliferation curve of MCF-7 cells transfected with DCTPP1 or control siRNAs. Values are mean ± SD (*n *≥ 3). **c** Cell cycle profile of DCTPP1-siRNA-transfected cells. Values are mean ± SD (*n *≥ 3). **d** Intracellular dNTP pool determination in DCTPP1-deficient cells. Values are mean ± SD (*n *≥ 3). **e** Down-regulation of DCTPP1 sensitizes cells to 5-FdUrd. MCF-7 cells (siCtrl and siDCTPP1) were exposed to increasing concentrations of 5-FdUrd for 24 h. The plot represents the relative population doubling (RPD) (± SD) of two independent experiments. **f** Western blot analysis of pyrimidine metabolism enzymes in DCTPP1-silenced cells. Anti-α-tubulin was used to normalize protein levels. Values are mean ± SD (*n *≥ 3)

DCTPP1 is a human NTP pyrophosphohydrolase with high specificity in vitro for dCTP [10]. Here, we sought to unveil in different cell types the role of the enzyme in the homeostasis of canonical nucleotide pools and cell viability and how it interrelates to other enzymes involved in pyrimidine metabolism. For this purpose, we accomplished silencing of DCTPP1 in MCF-7 cells by siRNA and analyzed in detail the consequences of this defect. DCTPP1 depletion was efficiently attained, 85–95% depletion (Fig. 1a) and induced a significant proliferation defect (40% growth inhibition) by day 8 post-transfection (Fig. 1b) similar to what has been previously described [13]. Likewise, cell cycle analysis of DCTPP1-knockdown MCF-7 cells revealed an increase in the percentage of cells in the G1 phase and a decrease in the proportion of cells in S and G2/M, indicating a delay in the progression of G1 to S, the latter being the active phase for dNTP biosynthesis (Fig. 1c and Supp. Figure 1). Pyrimidine dNTP pools were monitored (Fig. 1d) and an expansion of the dCTP pool was observed upon enzyme depletion (3.7 pmol/10^6^ control cells vs 5.5 pmol/10^6^ deficient cells). This result differs from what has been previously published for MCF-7 cells by Song et al. [13], who failed to detect an increase in the levels of intracellular dCTP. We ignore the reason for these differences, although it is possible that subtle differences in serum pyrimidine content may influence the results. Moreover, while intracellular levels of dGTP remained constant, MCF-7 cells depleted of DCTPP1 exhibited a significant reduction in the intracellular levels of dTTP (7.3 pmol/10^6^ control cells vs 6.4 pmol/10^6^ deficient cells) and the appearance of a substantial dUTP pool, absent in control cells (1.5 dUTP pmol/10^6^ cells). The presence of dUTP in combination with a decreased pool of dTTP can be highly mutagenic [17], since most DNA polymerases cannot distinguish between thymine and uracil, and the uracil/thymine incorporation ratio depends on the relative level of dUTP and dTTP. The ratios obtained here for cells lacking DCTPP1 implies that dUTP is incorporated instead of dTTP one out of five times, which represents a high risk of uracil accumulation in DNA. Thus, a novel role for DCTPP1 arises from these observations, suggesting that it is also involved in the homeostasis of other pyrimidine nucleotides.

An increased dUTP/dTTP ratio represents a critical factor in the cytotoxicity induced by chemotherapeutic agents that target thymidylate synthase (TS) [18]. To investigate whether the depletion of DCTPP1 sensitizes to TS inhibitors which further stimulate the expansion of the dUTP pool, we determined the effect of 5-fluoro-2′-deoxyuridine (5-FdUrd) on cell proliferation. As shown in Fig. 1e, down-regulation of DCTPP1 led to increased toxicity by 5-FdUrd at doses 1 nM and higher, suggesting that DCTPP1-deficient cells are more susceptible to chemotherapeutic drugs that promote imbalances of the nucleotide pool and alter the dUTP/dTTP ratio.

We next investigated how other enzymes involved in pyrimidine nucleotide homeostasis respond to perturbations in DCTPP1 expression and thus may contribute to the modifications observed in MCF-7 cells. As shown in Fig. 1f, deoxycytidine kinase (dCK), dCMP deaminase (DCTD) and dUTPase levels remained unaltered in DCTPP1-deficient cells while the levels of thymidine kinase 1 (TK1), an enzyme involved in thymidine (dThd) salvage, were reduced by approximately 50%. The decrease in TK1 could be partly related to the cell cycle alterations observed in these cells, since it is well documented that TK1 is subjected to cell cycle regulation. Thus G1 cells, which accumulate in the absence of DCTPP1, are largely deprived of TK1 due to a degradation process initiated at the end of mitosis by the anaphase promoting complex/cyclosome (APC/C) [19].

### DCTPP1-deficient MCF-7 cells exhibit increased uracil misincorporation and an activation of the DNA damage response

**Fig. 2:**
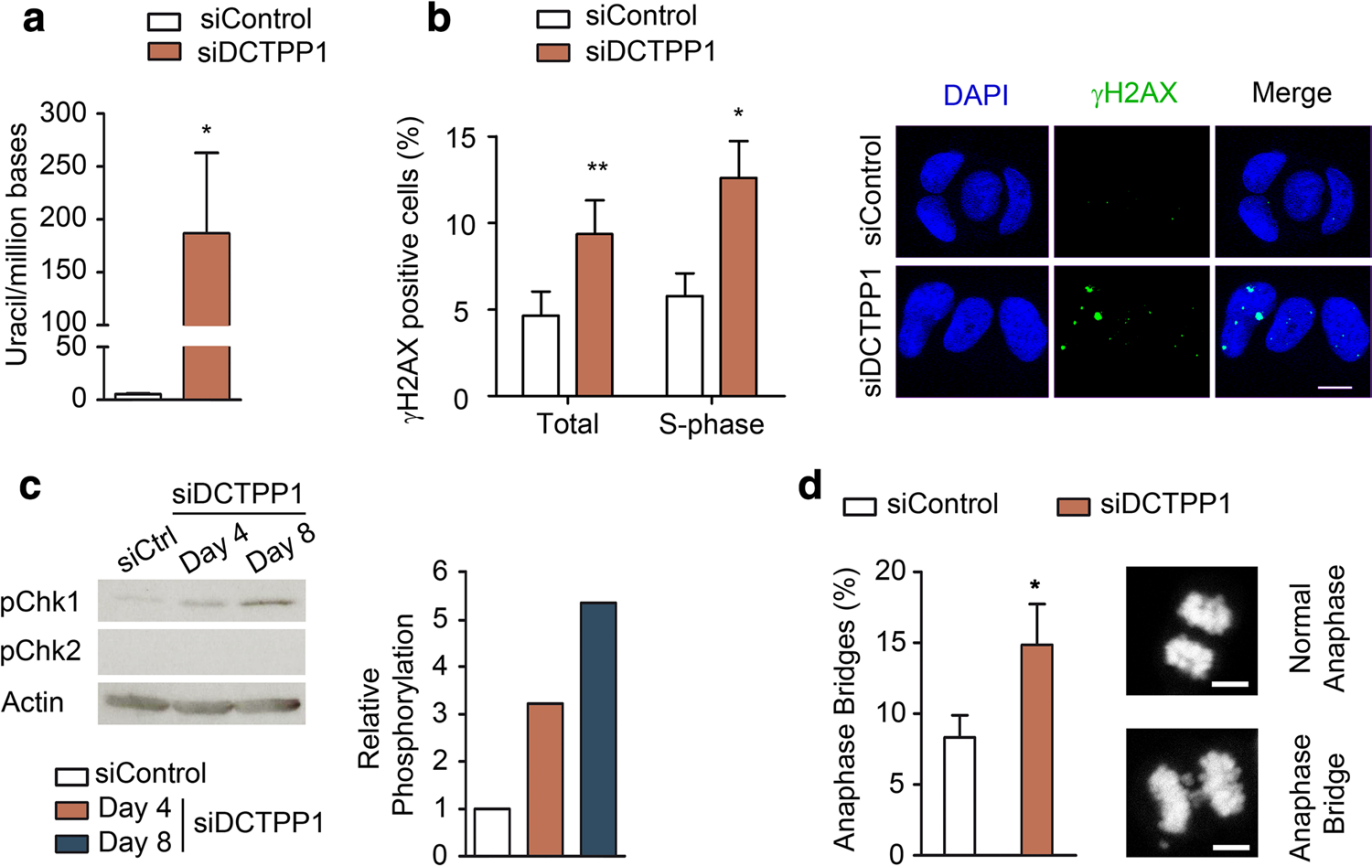
Down-regulation of DCTPP1 induces DNA damage in MCF-7 cells. **a** Determination of uracil in genomic DNA of MCF-7 cells transfected with control or DCTPP1 siRNAs. Values are mean ± SD (*n *≥ 3). **b** Immunofluorescence (IF) staining of γH2AX foci. Left panel, percentage of γH2AX-positive cells in total or S-phase populations after transfection with control or DCTPP1 siRNAs. Right panel, representative images of γH2AX staining. γH2AX-positive cells (≥ 5 foci/cell) were scored out of 100 cells per experiment (*n *= 3). **c** Western blot analysis of Chk1 and Chk2 phosphorylation in DCTPP1-depleted cells. Anti-actin was used to normalize protein levels. **d** Left panel, percentage of anaphase bridges in DCTPP1-silenced cells. Cells were synchronized for 18 h with nocodazole, released for 90 min and then fixed for DAPI staining. 100 mitoses were counted in each experiment (*n *= 3). Right panels, representative images of an anaphase bridge and a normal anaphase. Unless specified, all determinations were carried out at day 8 post-transfection. Scale bars indicate 10 μm

The elevated dUTP/dTTP ratio observed in cells devoid of DCTPP1 represents a substantial risk for uracil misincorporation into DNA and hence of genotoxic damage. We therefore assessed the presence of uracil in genomic DNA using a qPCR assay that utilizes two DNA polymerases with different capacity to replicate through uracil [20]. The difference in product formation gives an estimation of the uracil content in the DNA, yet the quantification is relative, as a zero must be established for the control sample. Notably, the uracil content of genomic DNA extracted from DCTPP1-silenced MCF-7 cells was significantly enhanced with regard to control siRNA-transfected cells, revealing a preferential accumulation of uracil in DCTPP1-depleted cells (186 uracils/million bases) (Fig. 2a).

Having demonstrated the modifications in base composition, we proceeded to evaluate the activation of the DNA damage response through assessment of γ-phosphorylation of the histone H2AX (H2AX phosphoserine-139), a hallmark of the cellular response to DNA double-strand breaks (DSBs). A significant twofold increase of the percentage of γH2AX-positive cells was detected when DCTPP1 expression was suppressed for 8 days (9.4% vs 4.9%), thus indicating the specific occurrence of DNA lesions in DCTPP1-deficient cells (Fig. 2b). To examine whether H2AX phosphorylation occurs specifically in cells performing DNA synthesis which are therefore susceptible to DSB formation, γH2AX foci were quantified by previous EdU-labeling of S-phase cells (Fig. 2b). Thus, when quantification was restricted to MCF-7 cells in the S-phase, an increased proportion of foci-containing cells was found in the absence of DCTPP1 (12.6% in siDCTPP1 vs 5.7% in siControl transfected cells). However, the number of γH2AX-positive cells in S-phase only represents a minor fraction of the total amount, thus suggesting that other mechanisms are involved in phosphorylation of H2AX.

Other key components of the DDR, the serine/threonine kinases Chk1 and Chk2, were also examined. As shown in Fig. 2c, DCTPP1 silencing specifically promotes the phosphorylation of Chk1, but not of Chk2 which is undetectable in MCF-7 cells. Furthermore, it has been previously described that replication stress causes chromosomal instability during mitosis [21]. Mitotic DCTPP1-depleted cells presented an increased percentage of anaphase bridges in comparison to control cells (14.8% vs 8.3% respectively) (Fig. 2d). All these observations together suggest the formation of double-strand breaks as one of the potential DNA lesions induced as a consequence of DCTPP1 depletion and reinforce the importance of DCTPP1 in maintaining genome stability.

### Modulation of the dUTP/dTTP ratio reduces genomic instability

**Fig. 3:**
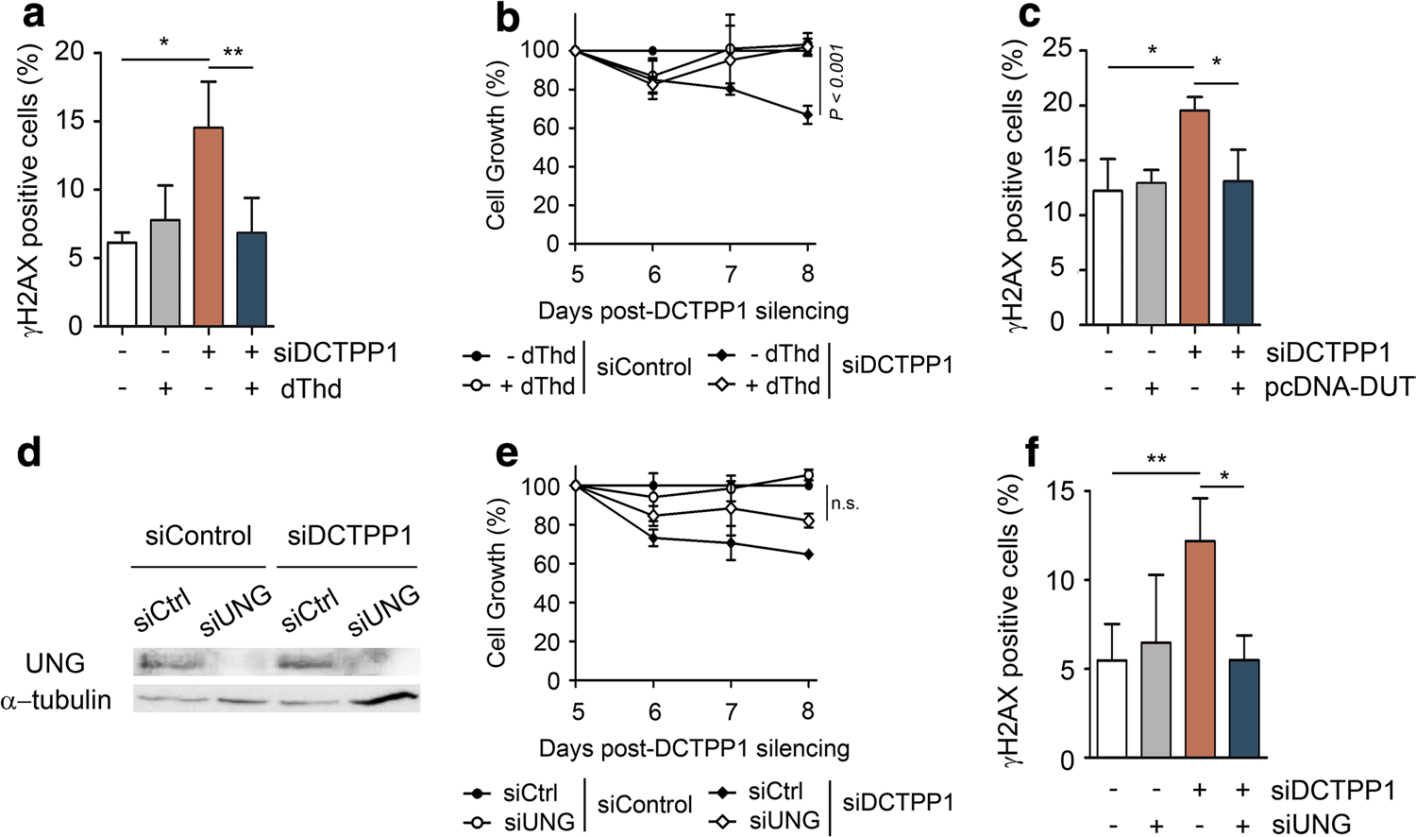
DNA damage response is abrogated by thymidine supplementation, dUTPase overexpression or uracil-DNA glycosylase suppression. **a** Quantification of γH2AX foci after thymidine (dThd) supplementation of DCTPP1 and control siRNA-transfected cells. At day 7 post-transfection, cells were supplemented with dThd (5 μM), incubated for 24 h and fixed for IF staining. Values are mean ± SD (*n *≥ 3). **b** Thymidine reverts the proliferation defect induced by DCTPP1 silencing. At day 5 post-transfection, control and DCTPP1-silenced cells were supplemented with dThd (5 μM) and cell growth monitored over a 4 day period. **c** Quantification of γH2AX foci after 48 h under dUTPase overexpression. At day 6 post-transfection, cells were retransfected with pcDNA-DUT or empty pcDNA plasmid. Values are mean ± SD (*n *≥ 3). **d** Western blot analysis of MCF-7 cells at day 4 post-transfection with UNG and control (siCtrl) siRNAs. Anti-α-tubulin was used to normalize protein levels. **e** Proliferation curves of MCF-7 cells transfected with DCTPP1, UNG or control siRNAs. Values are mean ± SD (*n *≥ 3). **f** Quantification of γH2AX foci after UNG silencing of DCTPP1-knockdown cells. Transfection with siUNG was carried out at day 4 post-transfection with siDCTPP1 or siCtrl and analyzed at day 8. Values are mean ± SD (*n *≥ 3). Unless specified, all determinations were carried out at day 8 post-transfection

Considering that uracil misincorporation might be the initial DNA damage that leads to the induction of γH2AX foci, we hypothesized that histone phosphorylation might be prevented by restoring the normal dUTP/dTTP ratio. Thus, cells transfected with control or DCTPP1 siRNA were cultured for 24 h in the presence of 5 μM of dThd before γH2AX analysis. The rationale behind this metabolic approach is that phosphorylation of thymidine via TK1 expands the synthesis of dTTP and restores an adequate dUTP/dTTP ratio. The incubation with dThd significantly decreased the percentage of γH2AX-positive cells in DCTPP1-knockdown cells (from 14.5% to 6.8% positive cells) indicating a cause–effect relationship between the dUTP/dTTP ratio and the levels of H2AX phosphorylation (Fig. 3a). Additionally, the cell growth delay induced by siRNA-mediated DCTPP1 silencing was reverted in the presence of thymidine (Fig. 3b).

To further investigate the implication of a high dUTP/dTTP ratio in genomic instability, human dUTPase was overexpressed in DCTPP1-silenced cells. Thus, cells previously transfected with either a control or a DCTPP1-siRNA, were retransfected with empty pcDNA or pcDNA-dUTPase (pcDNA-DUT) vectors. Indeed, the overexpression of dUTPase decreased the formation of γH2AX foci in a DCTPP1-deficient background (from 19.5 to 13% positive cells) (Fig. 3c) to levels very similar to those found in control cells transfected with pcDNA or with pcDNA-DUT (12.2% and 12.9%, respectively). The data support the hypothesis that the incorporation of dUTP during DNA replication is likely behind the activation of the DDR, a process than can be prevented by restoring an adequate dUTP/dTTP ratio either by dThd supplementation or by elimination of dUTP from the cellular pool.

Several studies have reported that the loss of uracil glycosylase activity (UNG) contributes to alleviate the deleterious phenotypes associated with increased dUTP/dTTP ratios [22–24]. We therefore set out to investigate the role of uracil-DNA glycosylase in DCTPP1-deficient MCF-7 cells. Strong down-regulation of UNG partially restored the proliferation of DCTPP1-silenced cells (from approximately 60–80% at day 8 post-transfection) (Fig. 3d, e). Moreover, the phosphorylation of H2AX was entirely abrogated upon UNG depletion (Fig. 3f). These results indicate that uracil removal and the subsequent generation of abasic (AP) sites appear to be the main source for the DSBs induced by DCTPP1 knockdown.

### Functional characterization of a DCTPP1 knockout HAP1 cell line

**Fig. 4:**
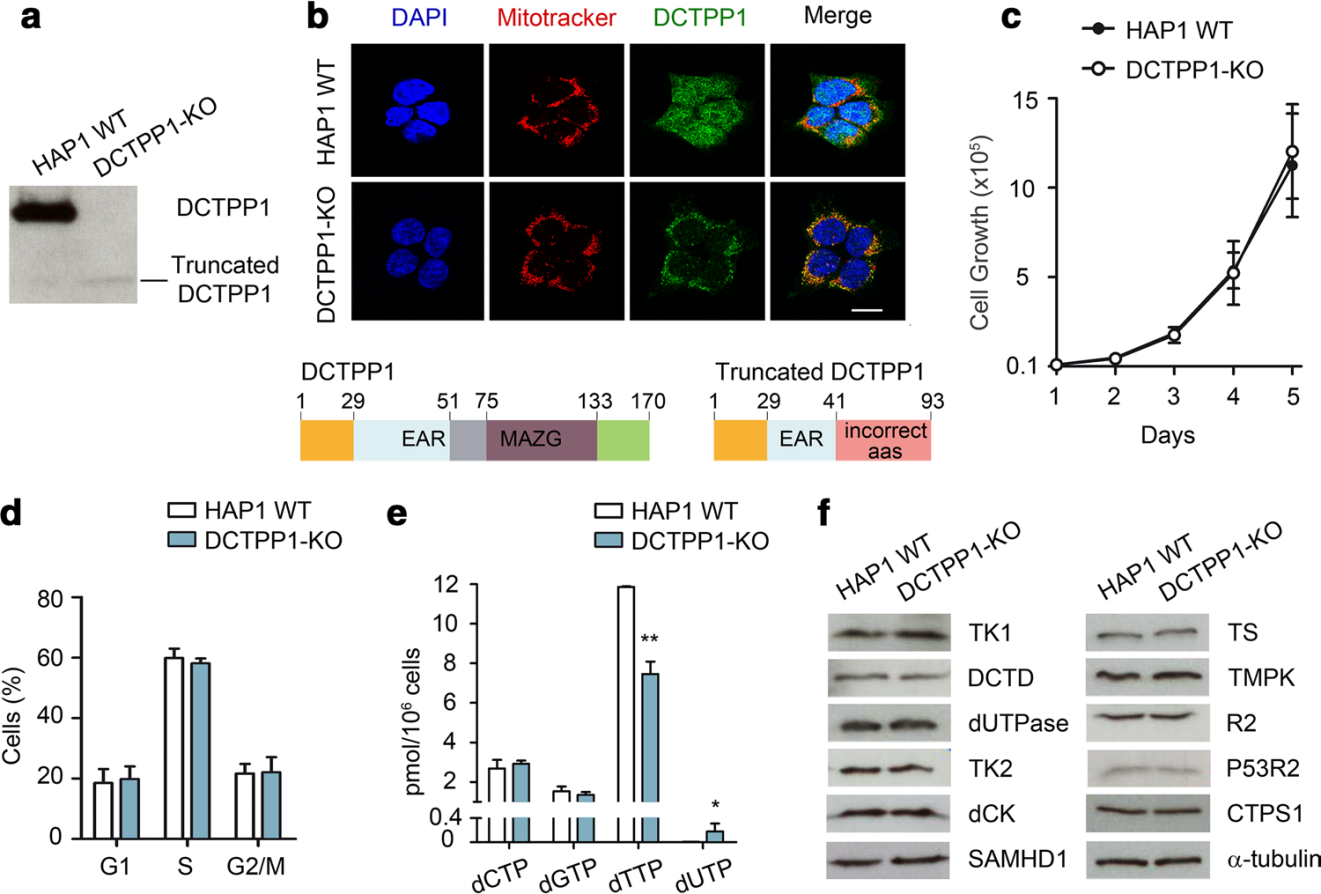
DCTPP1-knockout cells exhibit normal proliferation in spite of an imbalanced nucleotide pool. **a** Western blot showing the presence of a truncated non-functional DCTPP1 in the knockout cell line. **b** Immunofluorescence analysis showing the mitochondrial localization of the truncated DCTPP1 in DCTPP1-KO cells. Below, linear representations of the domains present in wild-type DCTPP1 and the truncated form resulting from CRISPR/Cas9 editing. **c** Proliferation curves of HAP1 WT and DCTPP1-KO cell lines. **d** Cell cycle analysis of DCTPP1-KO cells by FACS. **e** Determination of the pyrimidine dNTP pools. Values are mean ± SD (*n *≥ 3). **f** Comparison by western blot of the expression levels of different pyrimidine metabolism enzymes in parental HAP1 WT and DCTPP1-KO cells. Anti-α-tubulin was used to normalize protein levels. Results are mean ± SD (*n *≥ 3). Scale bars indicate 10 μm

While the down-regulation of DCTPP1 has profound consequences on cell cycle progression, nucleotide pools and DNA integrity in MCF-7 cells, a bona fide DCTPP1-knockout (DCTPP1-KO) cell line derived from the HAP1 cell line is readily available (Horizon Discovery). HAP1 is a near haploid human cell line that was derived from the male chronic myelogenous leukemia (CML) cell line KBM-7 [25]. To monitor the impact of the absence of DCTPP1 and identify factors contributing to tolerance to the lack of the enzyme, a viable clone harboring a 13 bp deletion in exon 2 of the DCTPP1 gene generated using the CRISPR/Cas9 technology was subjected to a functional characterization. These cells present a truncated form of DCTPP1 of approximately 10 kDa (Fig. 4a, b). Of the 93 amino acids of the truncated protein, only 41 correspond to the canonical sequence, the rest are aberrant amino acids (Fig. 4b). These cells lack a functional DCTPP1, since the resultant protein contains only one of the seven residues implicated in catalysis [26]. In contrast to MCF-7 cells, a deficiency in DCTPP1 does not affect either proliferation or cell cycle progression (Fig. 4c, d). A number of studies have reported phenotypic differences between knockout and knockdown approaches in different model organisms [27]. This phenotypic difference could be attributed to compensatory mechanisms triggered only after gene inactivation and clone selection. Indeed unexpectedly, DCTPP1 inactivation in HAP1 cells did not induce an expansion of the dCTP pool as described above for MCF-7. Here, the level of dCTP was estimated to be 2.6 pmol and 2.9 pmol (per 10^6^ cells) in control and DCTPP1-KO cells, respectively (Fig. 4e). In the latter, however, the pool of dTTP was again significantly reduced from 11.8 pmol/10^6^ to 7.4 pmol/10^6^, while a dUTP pool with potential genotoxic consequences was exclusively detected in deficient cells (0.25 pmol/10^6^ cells). No differences in the pool of dGTP were observed between HAP1 WT and DCTPP1-KO cell lines.

Changes in the expression of other key enzymes of pyrimidine metabolism which may be modulated in response to the inactivation of DCTPP1 were not detected (Fig. 4f). However, other types of enzymatic regulation can be responsible for the control of the dCTP pool in the absence of DCTPP1. On the other hand, TK1 levels were similar in both genetic backgrounds implying that it appears to be the loss of DCTPP1 and not differences in thymidine salvage, which was the major factor responsible for the decrease in dTTP. The results point toward a central role of DCTPP1 in the formation of dCMP for dTMP biosynthesis.

### The activation of the DNA damage response upon DCTPP1 inactivation can be reverted by the expansion of the dTTP pool or defects in uracil excision

**Fig. 5:**
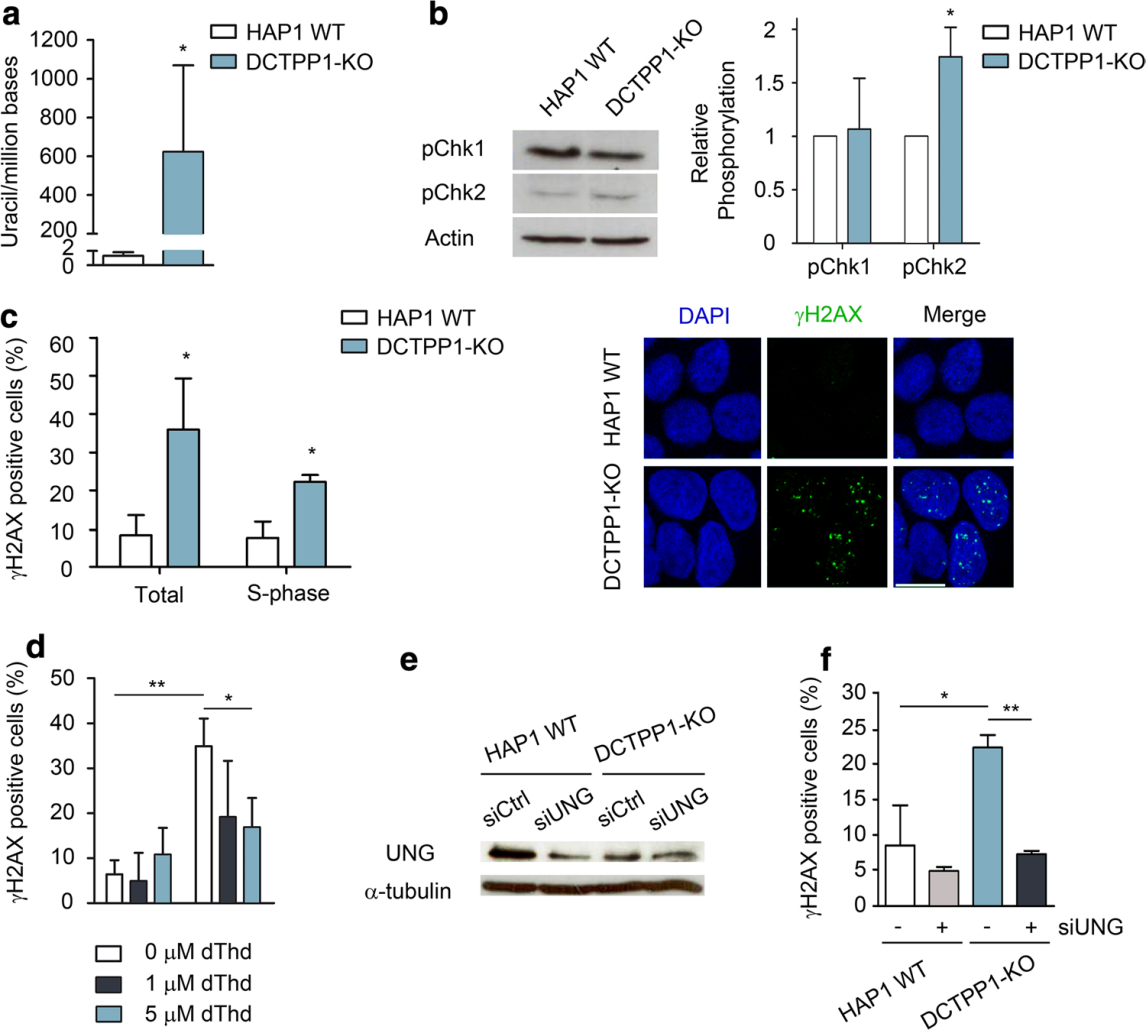
HAP1 cells defective in DCTPP1 present increased DNA damage. **a** Detection of uracil in genomic DNA of DCTPP1-KO cells. Genomic DNA isolated from HAP1 WT cells was used as a reference. **b** Western blot analysis of Chk1 and Chk2 phosphorylation. Anti-actin was used to normalize protein levels. **c** Left panel, percentage plots of γH2AX-positive HAP1 WT and DCTPP1-KO cells in total or S-phase populations. γH2AX-positive cells were scored out of 100 cells per experiment (*n *= 3). Right panel, representative images of γH2AX staining. Scale bars indicate 10 µm. **d** Determination of γH2AX foci after incubation of HAP1 WT and DCTPP1-KO cells with 1 or 5 μM of thymidine (dThd). **e** Western blot analysis of HAP1 cells at day 4 post-transfection with UNG and control (siCtrl) siRNAs. Anti-α-tubulin was used to normalize protein levels. **f** Quantification of γH2AX foci after UNG silencing in DCTPP1-knockout cells. Values are mean ± SD (*n *≥ 3)

Having established an expansion of the dUTP pool also in DCTPP1-KO cells, the relative uracil content was quantified again in control and DCTPP1-knockout genomic DNA samples (Fig. 5a). In correlation with the higher dUTP/dTTP ratio observed in DCTPP1-deficient cells, their DNA accumulates a higher amount of uracil (622 uracil per million bases more than parental cells). Consistently with their elevated genetic instability, HAP1 cells have easily detectable endogenous levels of pChk1, which appear sufficient to cope with further DNA damage resulting from DCTPP1 inactivation. pChk2 levels were slightly increased in DCTPP1-KO cells, suggesting that Chk2 phosphorylation may have a minor role in the DDR (Fig. 5b). On the other hand, the percentage of γH2AX-positive cells was significantly enhanced as a consequence of DCTPP1 inactivation: from 8.2 to 35.9% (data from total cell population) and from 7.6 to 22.2%, when considering only the S-phase population (Fig. 5c). When referred to the whole cell population, the total cells in the S-phase represent 4.5% and 12.9% respectively. Therefore, around one-third of γH2AX foci associated with DCTPP1 inactivation (12.9% out of 35.9%) were observed in S-phase cells and may be the result of slow or inefficient DNA repair during DNA replication.

To restore the dUTP/dTTP ratio, HAP1 WT and DCTPP1-KO cells were also treated with 1 µM or 5 µM of thymidine for 24 h before γH2AX foci evaluation. At these dThd concentrations, a thymidine block is not reached in HAP1 cells (Supp. Figure 2). In HAP1 WT cells, the percentage of foci was not significantly affected by the treatment with thymidine; in contrast, when DCTPP1-KO cells were supplemented with 1 µM or 5 µM dThd, the percentage of foci decreased from 34.9 to 26.3% and 16.8%, respectively, supporting the relationship between a high dUTP/dTTP ratio and H2AX phosphorylation as observed in MCF-7 cells (Fig. 5d). Likewise, silencing of UNG by siRNA fully reverted the H2AX phosphorylation observed in DCTPP1-KO cells (Fig. 5e, f), further reinforcing the notion that uracil repair intermediates are responsible for the activation of DDR in the absence of DCTPP1.

### DCTPP1-KO cells exhibit a hypermutator phenotype

**Fig. 6:**
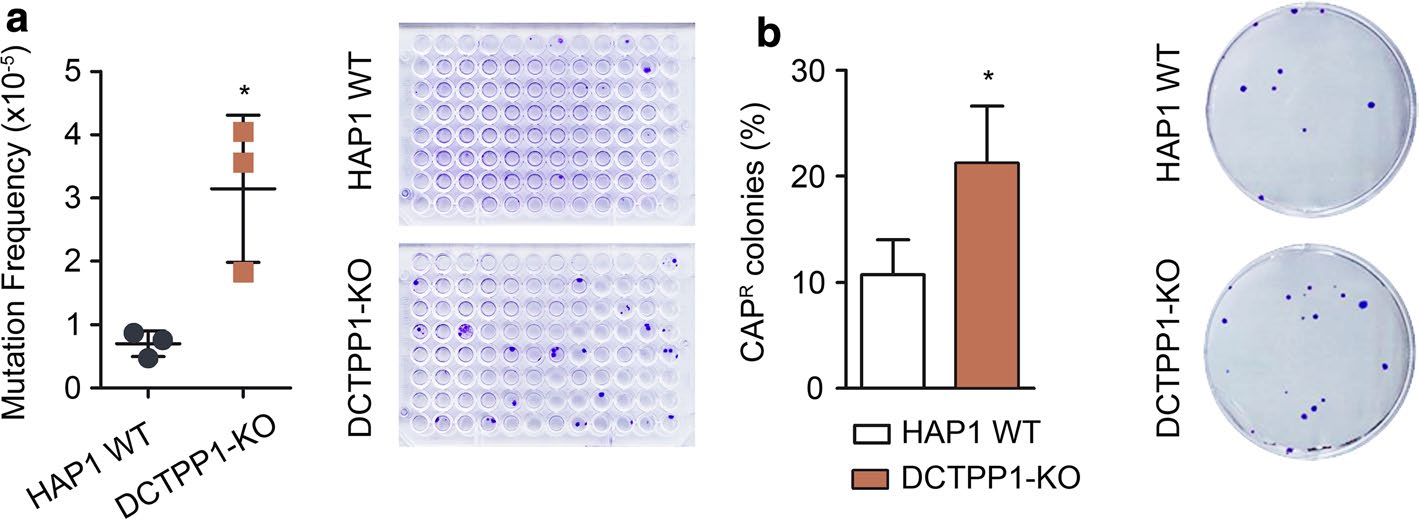
Nuclear and mitochondrial genomes from DCTPP1-deficient cells exhibit increased mutation frequencies. **a** Determination of hypoxanthine phosphoribosyltransferase (HPRT) gene mutation frequency. Cells were grown for 15 days and then treated with 6-thioguanine to select HPRT^−^ mutants. Three independent experiments are represented in the plot. **b** Chloramphenicol (CAP) resistance assay. The percentage of CAP-resistant colonies was calculated relative to the number of total viable colonies grown in the absence of CAP. Values are mean ± SD (*n *≥ 3)

The data presented suggest that the DCTPP1 mutant has to cope with severe genetic damage as a result of the alterations of the nucleotide pool. We have shown that cells defective in DCTPP1 are characterized by high amounts of uracil-DNA damage, increased anaphase bridge formation, cell cycle defects and the activation of DDR mechanisms. Hence, this mutant was expected to exhibit a spontaneous mutator phenotype. The haploid HAP1 cell line harbors only one copy of the hypoxanthine phosphoribosyltransferase (HPRT) gene, which can be used as a forward mutagenesis marker [28]. Under the conditions established, the spontaneous mutation frequency of DCTPP1-KO cells was 3.14 × 10^−5^, 4.5-fold higher than the value estimated for HAP1 WT cells (6.99 × 10^−6^) (Fig. 6a).

If the perturbation of the dNTP pool is at the origin of the mutator phenotype of DCTPP1-deficient cells, it should be reproduced in mitochondrial DNA, since its synthesis is greatly dependent on the cytosolic dNTP pool [29]. Besides, DCTPP1 is also present in the mitochondria and the mitochondrial dNTP pool might be disturbed in knockout cells. To assess mtDNA integrity, mutations at the 16S ribosomal RNA subunit encoded by the mtDNA were selected by resistance to chloramphenicol (CAP). Figure 6b shows the percentage of CAP-resistant colonies in WT and DCTPP1-KO cells lines. DCTPP1 deficiency strongly increased mtDNA mutagenesis from 10.7 to 21.2%. It is possible that DCTPP1 performs a specific mitochondrial role with an impact on mtDNA stability and that its mitochondrial activity is required for the homeostasis of the nucleotide pool in this compartment.

### Contribution of DCTPP1 to de novo dTTP synthesis in HAP1 cells

**Fig. 7:**
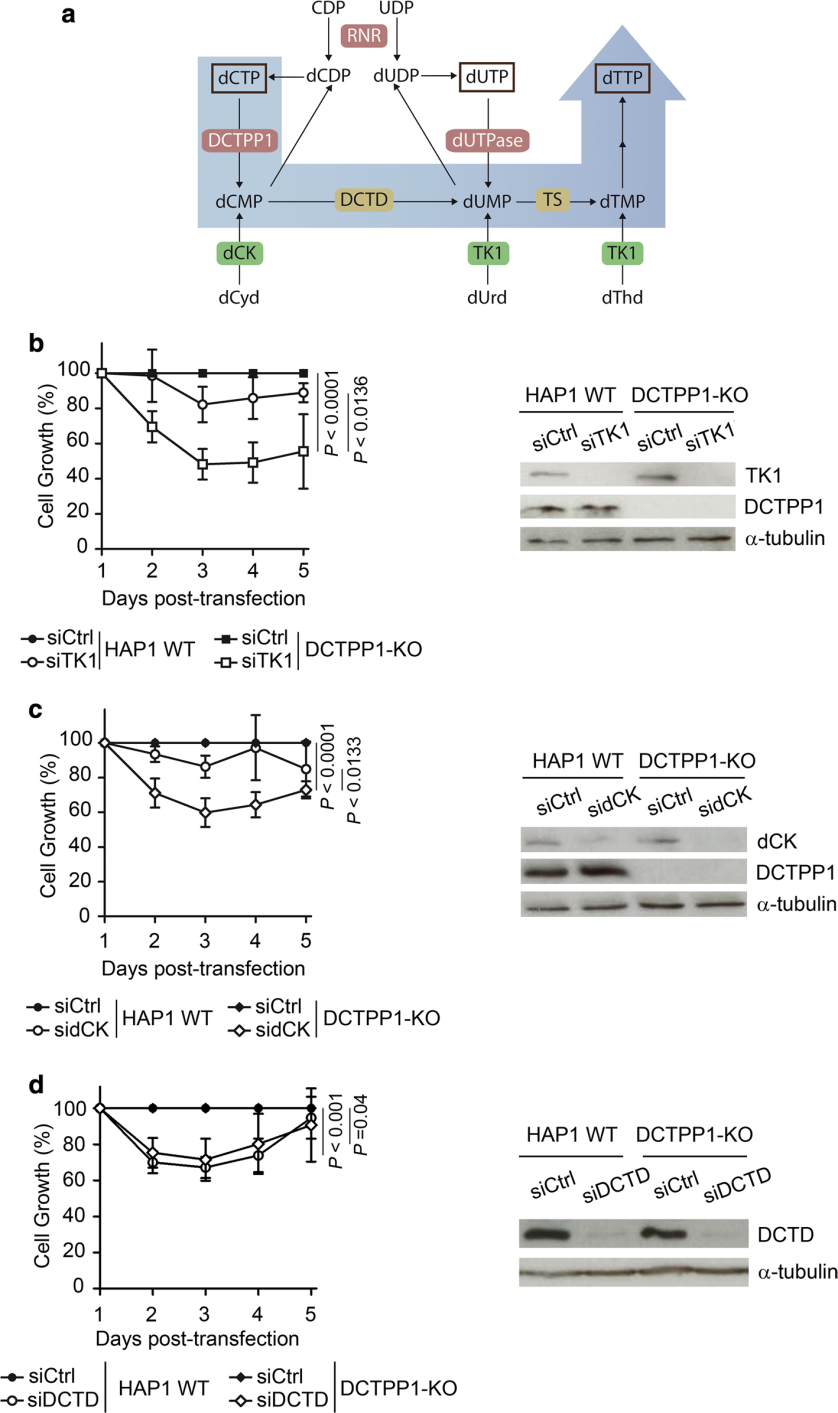
Nucleoside salvage is required for normal proliferation in the absence of DCTPP1. **a** Schematic diagram of the pyrimidine metabolic pathway in humans. In addition to its role in dCTP homeostasis, DCTPP1 is involved in the de novo synthesis of dTTP. The shaded blue arrow indicates the proposed route for the conversion of excess dCTP into dTTP. The DCTPP1 reaction product, dCMP, is converted to thymidylate (dTMP) through the consecutive reactions of dCMP deaminase (DCTD) and thymidylate synthase (TS). Red boxes: de novo synthesis enzymes; green boxes: enzymes involved in deoxynucleoside salvage; yellow boxes: enzymes involved in dTMP biosynthesis via dCMP. **b** Effect of thymidine kinase 1 (TK1) silencing on cell growth. Left panel, growth curve of HAP1 WT and DCTPP1-KO cells transfected with control or TK1 siRNAs. Right panel, analysis of protein levels at day 4 post-transfection. **c** Impact of deoxycytidine kinase (dCK) silencing on cell growth. Left panel, growth curve of HAP1 WT and DCTPP1-KO cells transfected with control or dCK siRNAs. Right panel, western blot analysis at day 4 post-transfection. **d** Down-regulation of dCMP deaminase (DCTD) in the presence or absence of DCTPP1. Left panel, growth curve of HAP1 WT and DCTPP1-KO cells transfected with control or DCTD siRNAs. Right panel, western blot analysis at day 4 post-transfection

The reduction of the dTTP pool upon DCTPP1 depletion indicates that the enzyme contributes in a relevant manner to de novo synthesis of dTTP. We hypothesized that this function is accomplished by providing dCMP which can be converted into dTMP in two consecutive reactions catalyzed by dCMP deaminase and thymidylate synthase activities. We therefore anticipated that in the absence of DCTPP1, thymidine salvage by TK1 and also deoxycytidine salvage by dCK might play a critical role in the synthesis and homeostasis of dTTP (Fig. 7a).

To address these questions, thymidine and deoxycytidine salvage were inhibited by siRNA. HAP1 WT and DCTPP1-KO cells were transiently transfected with either siRNA-TK1 or siRNA-dCK, and the loss of enzyme expression was verified by western blot. TK1 and dCK proteins were greatly decreased and the levels remained low until the end of the experiment (Fig. 7b, c). DCTPP1 knockout cells were hypersensitive to both TK1 and dCK depletion, with profound defects in cell growth (50–60% inhibition) at day 3 post-transfection. Conversely, TK1 or dCK silencing in HAP1 WT cells did not have a significant effect compared to control siRNA-transfected cells.

The relevance of dCMP deaminase in cell proliferation was also evaluated. HAP1 WT and DCTPP1-KO cells both present growth defects, yet are both equally sensitive to DCTD depletion by siRNA (Fig. 7d) suggesting that dCMP deamination is a crucial step in the provision of thymidylate and eventually dTTP. These results further support an important and novel role for DCTPP1 in the maintenance of the dTTP pool, such that in the absence of deoxycytidine or thymidine salvage, DCTPP1 becomes essential for cell viability.

### Nucleotide pools are strongly dependent on DCTPP1 and pyrimidine salvage

**Fig. 8:**
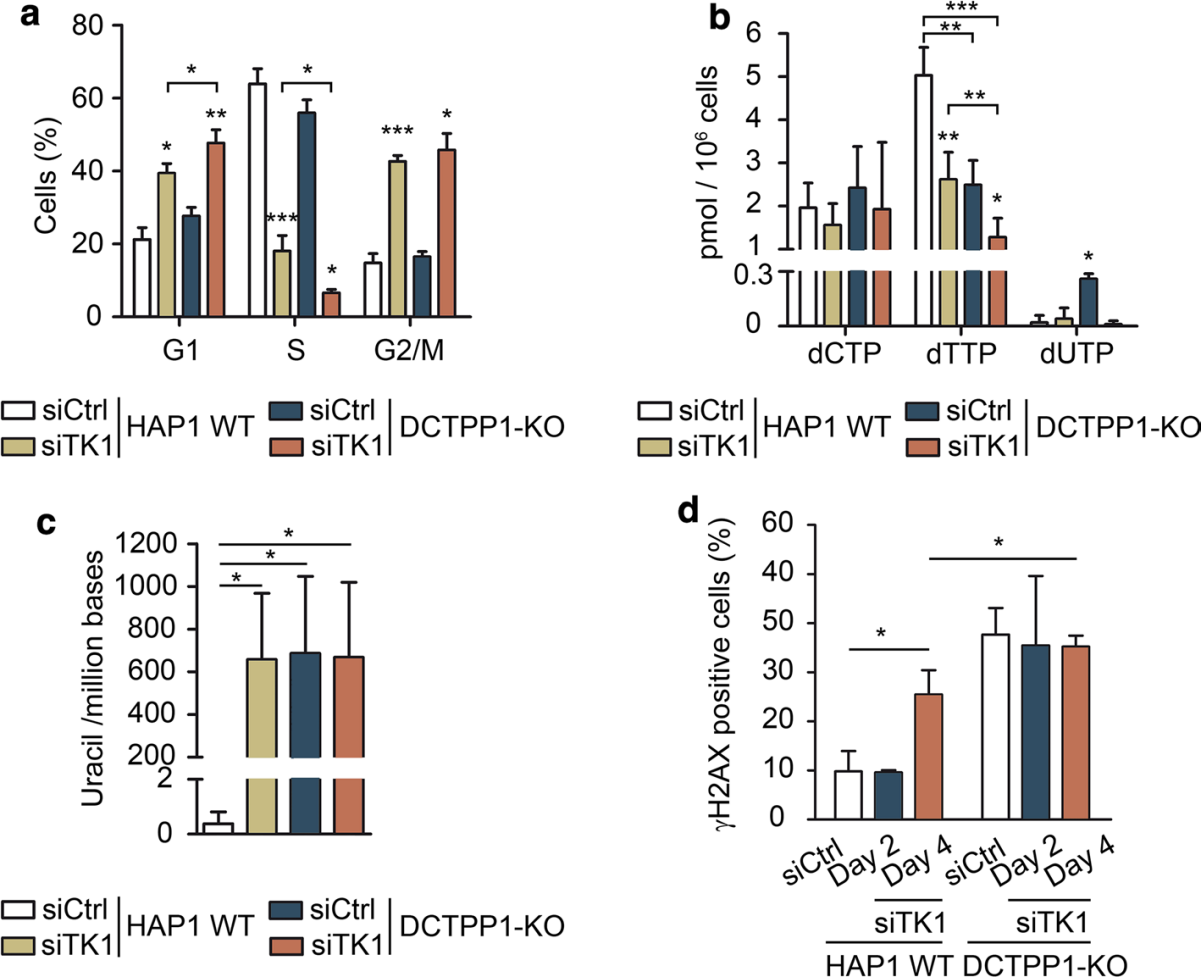
Effect of TK1 depletion on DCTPP1-knockout cells. **a** Cell cycle profile of HAP1 WT and DCTPP1-KO cells transfected with control or TK1 siRNAs at day 2 post-transfection. **b** Determination of the pyrimidine dNTP pools at day 2 post-transfection in HAP1 WT and DCTPP1-KO cell lines transfected with control or TK1 siRNAs. **c** Uracil in genomic DNA of HAP1 WT and DCTPP1-KO cells transfected with control or TK1 siRNAs at day 2 post-transfection. DNA isolated from HAP1 WT cells transfected with control siRNA was used as a reference. **d** Quantification of γH2AX foci. Percentage of γH2AX-positive cells was determined at days 2 and 4 post-transfection of HAP1 WT and DCTPP1-KO cells with control or TK1 siRNAs. γH2AX-positive cells were scored out of 100 cells per experiment (*n *= 3). All values are mean ± SD (*n *≥ 3)

The cell cycle was analyzed in TK1-silenced cells at day 2 post-transfection (Fig. 8a). In both, wild-type and DCTPP1-deficient backgrounds, the absence of TK1 caused a strong decrease in the number of cells in S-phase and simultaneous increases in both G1 and G2/M phases. These cell cycle anomalies induced by TK1 silencing, in particular the decrease in S-phase cells, were more pronounced in a DCTPP1-KO genetic background than in HAP1 WT cells (6.6% vs 18%, respectively), thus explaining their different proliferation rates.

The dNTP pool composition was also determined at day 2 after TK1 depletion (Fig. 8b). Again, no expansion of the dCTP pool was observed in DCTPP1-KO cells transfected with a siRNA control. Upon TK1 depletion, the dTTP pool in HAP1 WT cells was significantly reduced (from 5.0 to 2.6 pmol/10^6^ cells), reaching levels comparable to those of DCTPP1-KO cells (2.5 pmol/10^6^ cells). However, when TK1 was silenced in DCTPP1-KO cells, the dTTP levels were further severely diminished (1.3 pmol/10^6^ cells), evidencing the participation of both enzymes in the synthesis of dTTP.

The uracil levels in DNA from cells devoid of TK1 and DCTPP1 were high, but still comparable to those of cells with single enzyme defects in DCTPP1 or TK1 (Fig. 8c). On the other hand, the percentage of γH2AX foci in parental cells increased from 9.8% to 25.4% after 4 days of TK1 depletion, while DCTPP1-deficient cells showed massive immunostaining with the antibody (30–40% positive cells) before and after TK1 silencing (Fig. 8d). TK1 depletion in DCTPP1-KO cells does not result in increased DNA damage (Fig. 8c, d), although the decrease in the dUTP pool together with the reduction of the number of cells undergoing replication during the S-phase would minimize dUTP incorporation, and therefore may account for this latter observation.

## Discussion

Here, the metabolic function of DCTPP1 was studied by using two different reverse genetics approaches, transient gene silencing and CRISPR/Cas9-based gene targeting, in two established mammalian cell models: breast cancer MCF-7 cells and the KBM-7-derived HAP1 cell line, respectively. We observed that DCTPP1-knocked down MCF-7 cells showed an expanded dCTP pool, supporting a major role for DCTPP1 as a dCTP hydrolase. This observation contrasts with the data of Song et al. [13], who failed to detect changes in the pool of canonical nucleotides in the same cell type. By contrast, the level of this nucleotide remained unaffected in knockout HAP1 cells, where it is possible that a hitherto unknown compensatory response is induced to regulate dCTP levels. Conceivable mechanisms could include post-translational or allosteric regulation of SAMHD1 triphosphohydrolase [30, 31]. Indeed, phosphorylation of SAMHD1 threonine 592 appears to increase its dCTP hydrolase activity [32] and might compensate for the loss of DCTPP1. Moreover, modulation of CDP reduction, CDP or CTP biosynthesis could contribute to dCTP homeostasis in knockout cells.

Further analysis of the dNTP pool composition revealed a substantial reduction of dTTP and the presence of significant amounts of dUTP in both experimental models. These data suggest that DCTPP1 is not only implicated in keeping a balanced pool of dCTP, but also crucially contributes to the synthesis of dTTP. In support of this additional function for DCTPP1, we have shown that diminishing of nucleoside salvage by dCK or TK1 silencing reduces dTTP below critical levels and impairs proliferation of DCTPP1-deficient cells, indicating that in the absence of deoxycytidine or thymidine salvage, DCTPP1 becomes essential for the provision of thymidylate. In a previous study performed in HeLa and MRC-5 cells, down-regulation of DCTPP1 resulted in an expansion of the dCTP pool, while the levels of dTTP remained unchanged [10]. It is possible that the relative contribution of the two pathways to dTMP formation accounts for the differential growth phenotype and dNTP levels in different cell types.

A notable consequence of DCTPP1 depletion was uracil accumulation in DNA, activation of the DNA repair response and a hypermutator phenotype. Our results indicate that an elevated dUTP/dTTP ratio is the major cause of these observations. Consistently, we have shown that restoration of an adequate dUTP/dTTP ratio in DCTPP1-deficient cells, either by exogenous supplementation of thymidine or by up-regulation of dUTPase, reduced extensive genome damage marked by persistent γH2AX foci. Uncontrolled uracil incorporation provokes chromosomal instability and harmful effects in both prokaryotic and eukaryotic cells [33] mostly in an indirect manner, due to preferential misincorporation of dAMP or dCMP opposite the abasic site intermediate generated by the base excision repair pathway responsible for uracil elimination [23, 34]. Moreover, excessive dUTP incorporation leads to futile cycles of excision and synthesis that may eventually engender lethal DNA fragmentation due to replication fork collapse at uracil-excision intermediates [35]. In agreement with this model, a recent study has shown that an elevated uracil incorporation, promoted by the combination of high expression levels of the ribonucleotide reductase (RNR) subunit R2 and low levels of dUTPase, is related to increased accumulation of double-stranded breaks in AT-rich common fragile sites and a higher number of chromosomal fragile sites associated anaphase bridges [36].

In addition to restoring an adequate dUTP/dTTP ratio through thymidine supplementation or dUTPase overexpression, suppression of the main uracil glycosylase activity also alleviated the genotoxic phenotype associated with DCTPP1 depletion, thus indicating that uracil in DNA and the subsequent formation of AP sites is a major factor in the activation of the DDR. On the other hand, a detailed cell cycle analysis of histone H2AX phosphorylation revealed that a significant portion of the γH2AX-positive cells were outside S-phase and therefore DSBs in these cells were not caused by the collapse of the replication fork. In this sense, a number of studies have reported that the nucleotide excision repair (NER) activates the DNA damage response (including H2AX phosphorylation) in non-S-phase cells [37–39]. Indeed, although AP sites are not helix distorting lesions, they can be recognized and repaired by NER [40–42]. Thus, while DSBs may arise from multiple pathways, involving or not DNA replication, the excision of uracil by UNG and the generation of AP sites, if not repaired efficiently, may lead to further genotoxic damage.

The association of pyrimidine pool disequilibrium with replication stress and genome instability has been extensively documented. Specifically, fission yeast dCMP deaminase mutants exhibit a phenotype that is clearly reminiscent of the one described for MCF-7 cells after depletion of DCTPP1, consisting in perturbed dCTP (elevated) and dTTP (decreased) pools, activation of genome integrity checkpoints, impaired DNA replication and defective growth [43]. Moreover, cytidine deaminase (CDA) down-regulation and elevated dCTP pools have been associated to Bloom syndrome, a genetic rare disease produced by mutations in the BLM gene and characterized by defective DNA replication, high rates of sister chromatid exchange, a dramatic increase in the number of chromatin bridges and predisposition to cancer [44].

Here, we show that DCTPP1-depleted cells are hypersensitive to inhibition of de novo dTMP biosynthesis, yet several studies also point toward the pathophysiological relevance of DCTPP1. Thus, observations have been reported that suggest that DCTPP1 is under MYC regulation. For instance, stauprimide treatment, which suppresses MYC transcription in various cancer cell lines, induces selective down-regulation of MYC target genes including DCTPP1 [45]. DCTPP1 appears to be up-regulated in liver stem cells overexpressing MYC [46]. Overexpression of DCTPP1 has been also described in multiple tumors [15] and has been identified as a poor prognosis marker in gastric, breast and prostate cancer [13, 16, 47]. We propose that DCTPP1 should now also be considered as a major factor contributing to pyrimidine homeostasis. Remarkably, depletion of the enzyme not only increased the spontaneous mutation frequency of DCTPP1-KO cells, but also compromised the mtDNA integrity. Understanding cellular mechanisms for the preservation of mtDNA integrity is of paramount importance, because it can provide targets for clinical interventions aimed at prevention and treatment of human diseases related to mutations in mtDNA.

In summary, our results shed new light on the mechanisms that regulate dNTP homeostasis. We have identified DCTPP1 as a crucial element in the enzymatic network involved in de novo synthesis of pyrimidines that provides a novel pathway for the conversion of dCTP into dTTP. DCTPP1 initiates this route by generating dCMP that is subsequently channeled to the synthesis of thymidylate (Fig. 7a). A singular feature of nucleotide metabolism is the duplication of pathways; thus, we have seen upon inhibition of DCTPP1 that cells are highly dependent on nucleoside salvage. We have also established that a defect in DCTPP1 causes imbalance of pyrimidine nucleotide pools, a metabolic function that is intrinsically linked to the preservation of genome integrity. These observations are in accordance with recent studies showing that precise regulation of dNTP catabolism is a prominent factor to prevent replication stress, genome damage and cell death [9, 36]. Modulation of DCTPP1 activity might be a valuable tool to disrupt de novo synthesis and nucleotide homeostasis in highly proliferative cells with a great demand for DNA precursors such as cancer cells. Its role in mutations arising during processes such as neurodegeneration or aging may also be considered. In addition, previous work has shown that inhibition of DCTPP1 may improve the action of certain pyrimidine nucleoside analogs used as anticancer drugs [11, 12, 48, 49]. Finally, its role in the maintenance of mtDNA integrity may provide a new avenue for therapies of diseases related to nucleotide supply and balance in this organelle. Future studies will demonstrate the clinical utility of these approaches.

## Materials and methods

### Cell lines and siRNA transfections

The haploid cell lines HAP1 WT and HAP1 DCTPP1-KO (Catalog ID HZGHC003248c012) were purchased from Horizon Discovery. The HAP1 DCTPP1-KO cell line was genetically modified by CRISPR/Cas9 and contains a 13 bp deletion in exon 2. Wild-type and knockout cell lines were maintained in Iscove’s modified Dulbecco’s medium (IMDM) (Life Technologies) supplemented with 10% fetal bovine serum (FBS) (Life Technologies), 100 units/mL penicillin and 100 µg/mL streptomycin (Life Technologies). MCF-7 cells (HTB-22, ATCC^®^) were cultured in Eagle’s minimum essential medium (MEM) (Life Technologies) supplemented with 10% FBS, 100 units/mL penicillin and 100 µg/mL streptomycin.

Transient silencing was carried out with siRNA oligonucleotide pools (ON-TARGETplus smart pool, Dharmacon) specific for DCTPP1 [10], TK1, dCK, DCTD and UNG. TK1 was targeted with a siRNA pool with the following sequences: 5′- GGGCCGAUGUUCUCAGGAA-3′; 5′-GCAUUAACCUGCCCACUGU-3′; 5′-GCACAGAGUUGAUGAGACG-3′ and 5′-CAAAGACACUCGCUACAGC-3′. The siRNA pool directed against dCK consisted of the following sequences: 5′-CCAGAGACAUGCUUACAUA-3′; 5′-AAAGCUGGCUCCUGCAUAG-3′; 5′-UAUCAAGACUGGCAUGACU-3′ and 5′-GGAAUGUUCUUCAGAUGAU-3′. The siRNAs directed against DCTD consisted of the following sequences: 5′-GCAAGAAACGGGACGACUA-3′; 5′-CCUUGUAAUGAAUGCGUA-3′; 5′-UCAAUUAACAGCAGACCGA-3′ and 5′-GGGGUGACAUUCCGGAAAU-3′. To target UNG mRNA the siRNA pool included the following siRNAs: 5′-UUAUCAAGCUAAUGGGAUU-3′; 5′-GAACUCGAAUGGCCUUGUU-3′; 5′-GAAGCGGCACCAUGUACUA-3′ and 5′-UAUAGAGGGUUCUUUGGAU-3′. The negative control comprised four non-targeting siRNA oligonucleotides (ON-TARGETplus non-targeting pool, Dharmacon). Transfection was performed using jetPRIME^®^ (Polyplus) for HAP1 cells and Lipofectamine 2000 (Life Technologies) for MCF-7 cells according to the manufacturer´s instructions.

### Proliferation assays

To perform the proliferation assays, cells were seeded into 96-well plates (2 × 10^3^ cells/well) 24 h after transfection. Every 24 h, cells were incubated with 20 µL of Resazurin (1.1 mg/mL (Sigma-Aldrich) in the dark for 2 h at 37 °C. Cell growth was determined by measuring the fluorescence at 570 nm (excitation wavelength) and 590 nm (emission wavelength) in a SpectraMax GEMINI EM microplate reader (Molecular Devices).

SiRNA-transfected cells (MCF-7) were seeded into 96-well plates (2 × 10^3^ cells/well) at day 5 post-transfection. 48 h after seeding, cells were exposed to increasing doses of 5-fluoro-2′-deoxyuridine (5-FdUrd) (0-1 μM) (Sigma-Aldrich) for 24 h. For thymidine (dThd) supplementation, siRNA-transfected cells were seeded at day 5 post-transfection and 5 μM of dThd was added to the culture medium and renewed every 24 h. The relative population doubling (RPD) was calculated as PDtreated/PDuntreated × 100%, where PD = {log [N24h/N0]}/log 2 [50].

### Cell cycle analysis by fluorescence-activated cell sorting (FACS)

For cell cycle analysis, cells were pulse labeled with 10 µM BrdU (Becton–Dickinson) for 1 h at 37 °C. After BrdU incubation, cells were trypsinized, washed with 1 × PBS and then fixed and permeabilized with 70% ice-cold ethanol overnight. After fixation, cells were washed (1 × PBS and 0.2% Tween 20), incubated 20 min with 4 M HCl and 1% Triton X-100, washed three times and then incubated at 4 °C overnight with anti-BrdU-FITC antibody (Becton–Dickinson) diluted 1:5 in 1% Blocking Reagent (Roche). For DNA staining, cells were incubated with 0.05 mg/mL of propidium iodide and 0.05 mg/mL of RNAse at room temperature for 20 min. Finally, cells were analyzed using a FACSAria III cell sorter flow cytometer (Becton–Dickinson). Data were evaluated using FlowJo cell analysis software.

### Immunoblotting and antibodies

Cell pellets were incubated with RIPA buffer (25 mM Tris:HCl pH 7.6, 150 mM NaCl, 1% NP-40, 1% sodium deoxycholate, 0.1% SDS) (Sigma-Aldrich), containing 1 × Halt Protease/Phosphatase Inhibitors (Thermo-Fisher Scientific) and incubated 10 min on ice. Lysates were centrifuged at 14,000*g* for 15 min at 4 °C and the supernatant stored at − 20 °C. Extracts were mixed with SDS-loading buffer, boiled, electrophoresed and transferred to PVDF membranes. Primary antibodies were incubated at 4 °C overnight.

The antibodies anti-DCTPP1 and anti-dUTPase are anti-rabbit polyclonal antibodies generated in our laboratory [10, 11]. Antibodies directed against TK1 (C-4), dCK (H-3), DCTD (F-9), TMPK (B-8), TS (H-265) and R2 (A-5) were obtained from Santa Cruz; p53R2 (Sigma-Aldrich); SAMHD1 (ABIN3187981) from Antibodies Online; TK2 (Acris); pChk2-Thr68 (C13C1) and pChk1-Ser345 (133D3) from Cell Signaling technology; CTPS1 (Abcam); α-tubulin (B-5-1-2) and actin (MM2/193) from Sigma-Aldrich and anti-UNG (GeneTex).

### dNTP pool size determination

Briefly, 2.2 × 10^6^ cells were extracted with 1.2 mL of 1:1 (v:v) methanol/water at − 20 °C and the suspension was vortex mixed. Samples were then subjected to two freeze–thaw cycles (10 min each at dry ice/ethanol) and then centrifuged at 16,000*g* for 20 min and 4 °C. The supernatants were collected, split into three 250 µL aliquots for the determination of dCTP, dTTP, dUTP and dGTP and stored at − 80 °C. Samples were dried under vacuum and then dissolved in 40 µL of buffer (34 mM Tris/HCl pH 7.8 and 5 mM MgCl_2_) with or without 30 ng of dUTPase and incubated for 20 min at 37 °C. The reaction was stopped with 100% of cold methanol and centrifuged at 16,000*g* for 20 min at 4 °C, dried and used for the quantification of the dNTP pool as previously described [10].

### Determination of uracil in DNA

The incorporation of uracil into genomic DNA was determined using a quantitative real-time PCR-based assay following the protocol described [20, 51]. Briefly, genomic DNA was extracted and digested with SacI-HF (New England Biolabs), and 3–5 kb fragments containing the target template, GAPDH gene, were isolated and purified from 1% agarose gel. A series of twofold dilutions was amplified with PfuTurbo Hotstart (Agilent Technologies) or Taq (Bioline) DNA polymerases as previously described [11]. All values refer to wild-type or control siRNA-transfected cells.

### Immunofluorescence analysis

For γH2AX foci analysis, cells were first seeded on sterile glass coverslips in 12-well plates. Cells were fixed and permeabilized as previously described [11], incubated with 1:400 diluted polyclonal anti-γH2AX antibody (Abcam) and detected with secondary Alex Fluor^®^ 488-conjugated goat anti-mouse IgG (Life Technologies). For EdU staining, cells were pulse labeled with 10 μM EdU (Invitrogen) prior to cell fixation. EdU incorporation was detected with the Click-iT^®^ EdU AlexaFluor^®^ 594 imaging kit (Invitrogen). Digital images were captured using a LEICA TCS SP5 confocal microscopy system and analyzed using Fiji software.

To visualize DCTPP1, HAP1 WT and DCTPP1-KO, cells were grown on sterile glass coverslips. For mitochondrial labeling, cells were incubated with 250 nM of MitoTrackerTM Red CMX Ros (Invitrogen) for 30 min at 37 °C. Fixation, permeabilization and blocking were performed as previously described. Cell were incubated with anti-DCTPP1 at a 1:1000 dilution for 1 h, washed and incubated with Alexa Fluor^®^ 488-conjugated anti-rabbit secondary antibody. Digital images were captured using a LEICA TCS SP5 confocal microscopy system.

### Quantification of anaphase bridges

MCF-7 cells were seeded on sterile glass coverslips and synchronized at G2/M by incubation with 50 ng/µL of nocodazole (Sigma-Aldrich) for 18 h. Cells were then incubated in normal MEM medium for 90 additional minutes before being fixed and DAPI stained. Digital images were captured using a LEICA TCS SP5 confocal microscopy system.

### Hypoxanthine-guanine phosphoribosyltransferase (HPRT) mutation assay

HAP1 WT and DCTPP1-KO cell lines were initially grown for 3 days in IMDM complete medium supplemented with 1 × HAT (hypoxanthine–aminopterin–thymidine) (Life Technologies). HPRT-null pre-existing mutants were removed by blocking de novo purine biosynthesis with aminopterin. Following HAT treatment, cells were incubated with HT (hypoxanthine–thymidine) (Life Technologies) for 24 h to restore de novo metabolism. Cells were passaged every 2–3 days and grown for 14 days prior to 6-thioguanine (6-TG) (Sigma-Aldrich) selection to fix spontaneous HPRT^−^ mutations. Cells were then seeded into 96-well plates (10,000 cells/well) and grown for 14 days in the presence of 2 µM 6-TG, which was renewed every 2–3 days. To determine plating efficiency, 1 cell/well was seeded into a 96-well plate, in non-selective medium and grown for 14 days. After 6-TG selection, colonies were fixed with a 3:1 methanol:acetic acid solution and stained with a solution of 0.5% of crystal violet in methanol. Plating efficiency and mutation frequencies were determined as previously described [28].

### Chloramphenicol resistance assay

Chloramphenicol (CAP) resistance was determined as a measure of mtDNA mutagenesis. HAP1 WT and DCTPP1-KO cells were seeded onto six-well dishes at 300 cells/well. 24 h after seeding, 300 µg/mL of CAP (Sigma-Aldrich) was added to each well and cells were grown in CAP-containing medium for 7 more days. To determine the plating efficiency, the same number of cells were seeded and cultured in the absence of CAP. Cells were allowed to grow for 7 more days after CAP treatment and then fixed with methanol:acetic acid (3:1 v/v) for 5 min and stained with 0.5% crystal violet solution. Colonies were counted using an optical microscope. The proportion of CAP-resistant colonies was calculated by dividing the number of colonies formed after CAP treatment by the number of colonies formed in the absence of CAP.

### Statistics

Student’s *t* test was used to compare different sets of data. Two-way ANOVA was used to analyze normally distributed data, followed by Dunnett’s post hoc test. Statistics were calculated with IBM SPSS Statistics or GraphPad Prism 5. Results are expressed as mean ± SD of at least three independent experiments. *P < 0.05, **P < 0.01, ***P < 0.001.

### Electronic supplementary material

Below is the link to the electronic supplementary material.

**Supplementary Fig.** **1.** BrdU vs PI plots of representative siControl and siDCTPP1 samples corresponding to data from Fig. 1c (TIFF 11471 kb)

**Supplementary Fig.** **2.** Culture supplementation with 1 and 5 μM of thymidine does not induce thymidine block. Cell cycle progression was analyzed in HAP1 WT and DCTPP1-KO cells after 24 h incubation with 1 or 5 μM of thymidine (dThd). Results are mean ± SD (n ≥ 3) (TIFF 9983 kb)
