# Supplementary figures and images for: DCTPP1 prevents a mutator phenotype through the modulation of dCTP, dTTP and dUTP pools
Source: Cell Mol Life Sci. 2019 Aug 3;77(8):1645–60. doi: 10.1007/s00018-019-03250-x (PMC7162842; doi:10.1007/s00018-019-03250-x)

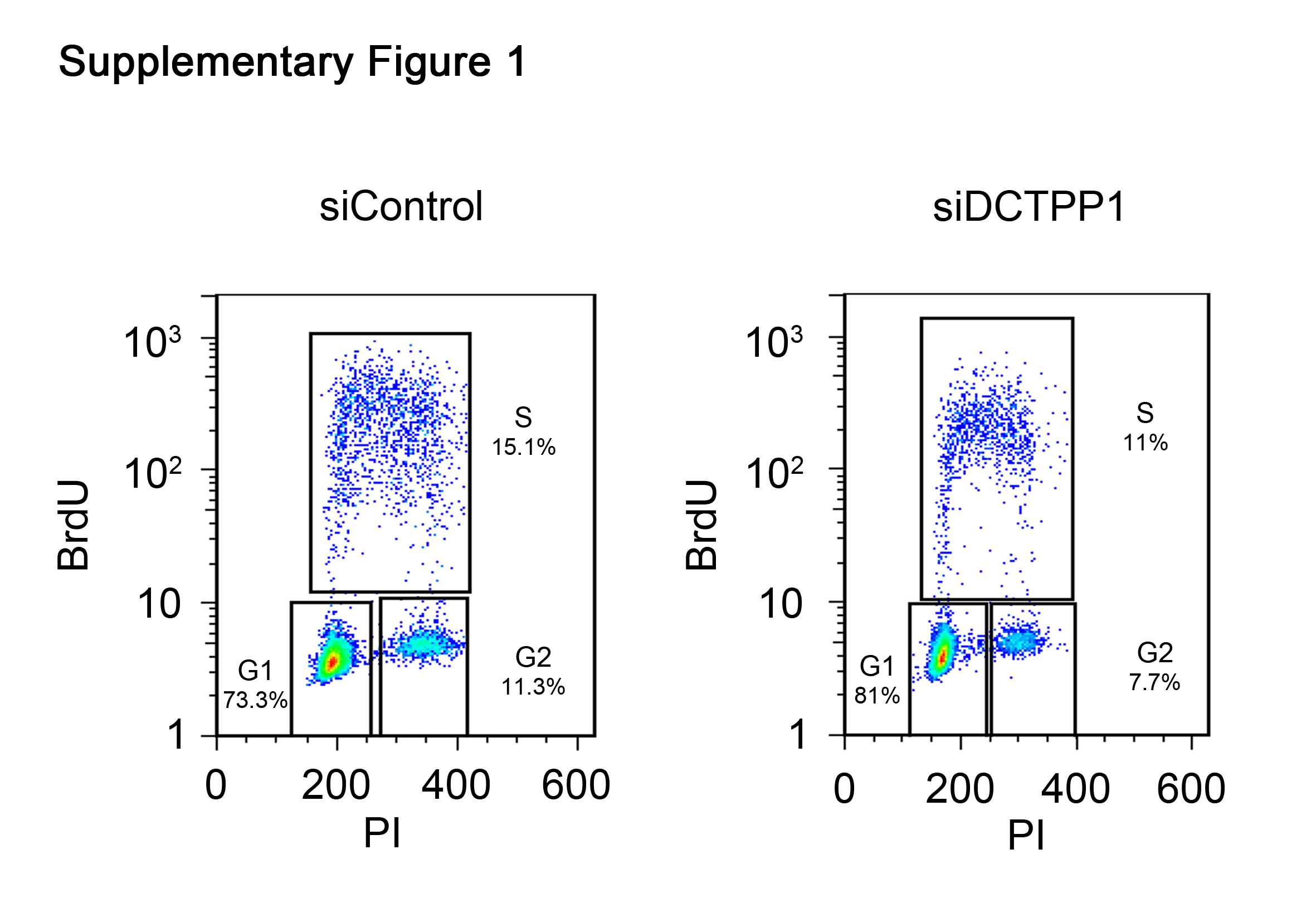

Supplement: Supplementary file 1 — Supplementary Fig. 1. BrdU vs PI plots of representative siControl and siDCTPP1 samples corresponding to data from Fig. 1c (TIFF 11471 kb) [file 18_2019_3250_MOESM1_ESM.tif]

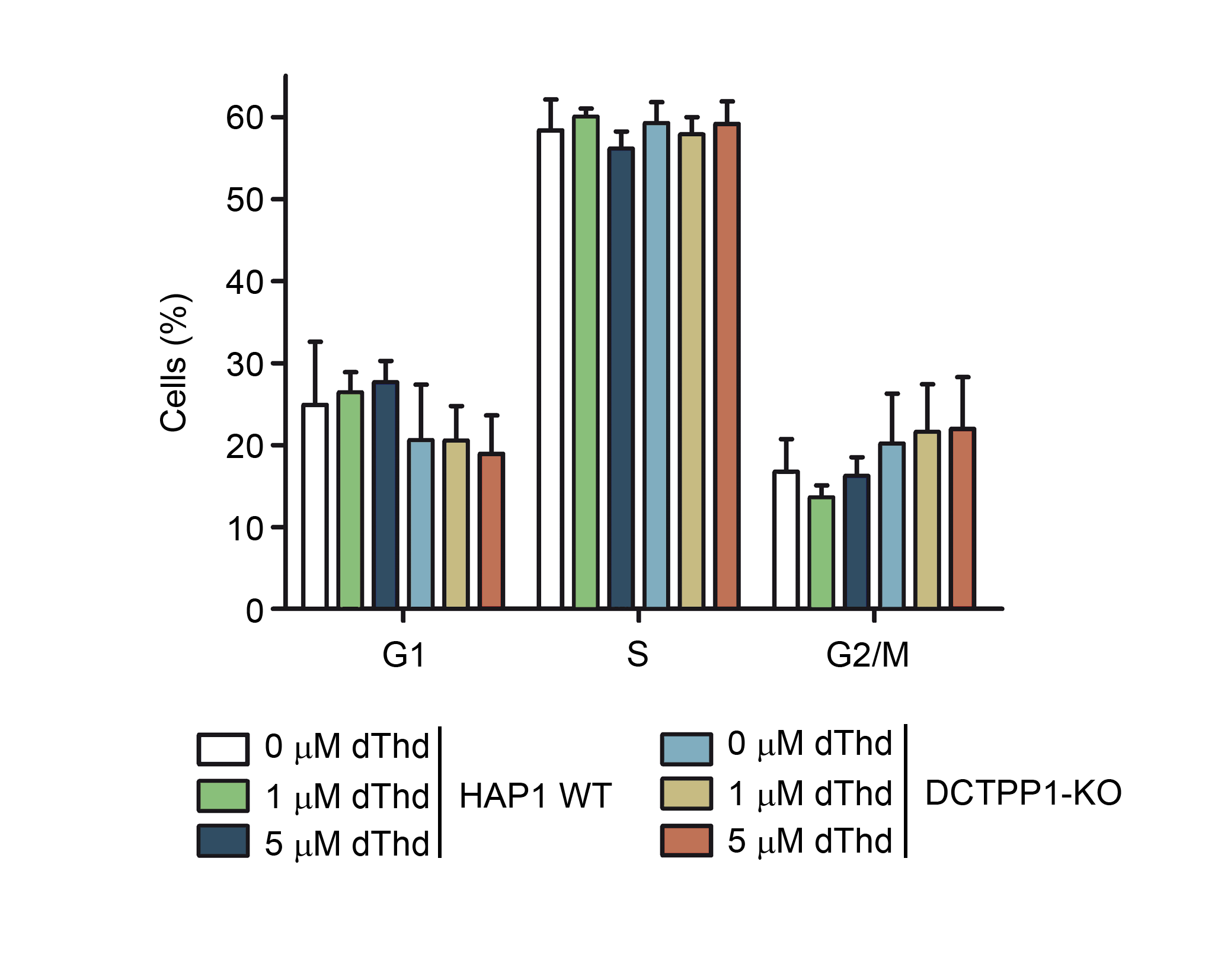

Supplement: Supplementary file 2 — Supplementary Fig. 2. Culture supplementation with 1 and 5 μM of thymidine does not induce thymidine block. Cell cycle progression was analyzed in HAP1 WT and DCTPP1-KO cells after 24 h incubation with 1 or 5 μM of thymidine (dThd). Results are mean ± SD (n ≥ 3) (TIFF 9983 kb) [file 18_2019_3250_MOESM2_ESM.tif]
